# Evaluation of the Minimum Sampling Design for Population Genomic and Microsatellite Studies: An Analysis Based on Wild Maize

**DOI:** 10.3389/fgene.2020.00870

**Published:** 2020-09-18

**Authors:** Jonás A. Aguirre-Liguori, Javier A. Luna-Sánchez, Jaime Gasca-Pineda, Luis E. Eguiarte

**Affiliations:** ^1^Departamento de Ecología Evolutiva, Instituto de Ecología, Universidad Nacional Autónoma de México, Mexico City, Mexico; ^2^Department of Ecology and Evolutionary Biology, UC Irvine, Irvine, CA, United States

**Keywords:** genomics of populations, landscape genomics, local adaptation, massive parallel sequencing, Mexican wild maize, sampling design

## Abstract

Massive parallel sequencing (MPS) is revolutionizing the field of molecular ecology by allowing us to understand better the evolutionary history of populations and species, and to detect genomic regions that could be under selection. However, the economic and computational resources needed generate a tradeoff between the amount of loci that can be obtained and the number of populations or individuals that can be sequenced. In this work, we analyzed and compared two simulated genomic datasets fitting a hierarchical structure, two extensive empirical genomic datasets, and a dataset comprising microsatellite information. For all datasets, we generated different subsampling designs by changing the number of loci, individuals, populations, and individuals per population to test for deviations in classic population genetics parameters (*H*_*S*_, *F*_*IS*_, *F*_*ST*_). For the empirical datasets we also analyzed the effect of sampling design on landscape genetic tests (isolation by distance and environment, central abundance hypothesis). We also tested the effect of sampling a different number of populations in the detection of outlier SNPs. We found that the microsatellite dataset is very sensitive to the number of individuals sampled when obtaining summary statistics. *F*_*IS*_ was particularly sensitive to a low sampling of individuals in the simulated, genomic, and microsatellite datasets. For the empirical and simulated genomic datasets, we found that as long as many populations are sampled, few individuals and loci are needed. For the empirical datasets, we found that increasing the number of populations sampled was important in obtaining precise landscape genetic estimates. Finally, we corroborated that outlier tests are sensitive to the number of populations sampled. We conclude by proposing different sampling designs depending on the objectives.

## Introduction

Massive parallel sequencing (MPS) has revolutionized the fields of molecular ecology, population genetics, and landscape genetics (Metzker, 2010; Stapley et al., 2010; Ekblom and Galindo, 2011). By increasing the number of polymorphic sites, it is now possible to estimate, with higher resolution, the genetic diversity, genetic structure, and demographic history of populations (Allendorf et al., 2010; Schoville et al., 2012; Excoffier et al., 2013; van Meier et al., 2017; Aguirre-Liguori et al., 2019a), and the environmental and geographic mechanisms that determine the connectivity between populations (Bradburd et al., 2013). MPS also allows for identifying genomic regions that could be under selection (Foll and Gaggiotti, 2008; Coop et al., 2010; Stapley et al., 2010; De Villemereuil and Gaggiotti, 2015).

MPS is powerful in detecting patterns of local adaptation and understanding how the environment structures genetic diversity; nevertheless, its potential capacity depends on sampling a large geographic area, and encompassing an adequate environmental and genomic representation of the species (Schoville et al., 2012; De Mita et al., 2013; Tiffin and Ross-Ibarra, 2014). Unfortunately, for many research groups MPS is still expensive, or in some other cases, such as rare or endangered species, obtaining a large number of populations or individuals, and/or enough DNA for genomic studies can be challenging. In addition, the bio-informatic processing required for large samples can be limiting, making it difficult to obtain adequate genomic representation for enough individuals and populations. A solution has been to prioritize sequencing power to compensate for fewer individuals or populations (Schiffels and Wang, 2020). However, in the context of local adaptation, sampling populations in different parts of the distribution or different environments can affect the adequate estimation of genetic parameters (Meirmans, 2015). For instance limited sampling can make it difficult identifying center-edge patterns (Eckert et al., 2008), or the detection of outlier regions that might be under selection (De Mita et al., 2013). Thus, it is crucial to determine the potential biases associated with sampling (number of individuals, loci, and populations) and to define the tradeoff between the sampling effort and the number of polymorphic regions obtained with MPS that are needed to obtain robust estimates (Pruett and Winker, 2008; Willing et al., 2012; De Mita et al., 2013; Fumagalli, 2013).

So far, different studies have evaluated the errors and biases generated in estimates of genetic parameters when a different number of populations, the number of polymorphic sites, and the number of individuals are used (Table 1 summarizes a list of studies that have evaluated sampling designs on population genetics studies). In summary, these studies have shown that parameters of mean genetic diversity (*F*_*ST*_, *F*_*IS*_, *H*s) are not affected by sampling a different number of loci, the number of individuals or the number of populations; however, the variance decreases as the number of populations, individuals, and loci increases (see summaries and references in Table 1). In contrast, these studies have shown that patterns of isolation by distance and isolation by environment (IBE) across reduced areas are sensitive to the number of populations sampled and the sampling design (linear, aggregated, random sampling).

**TABLE 1:** Summary of 19 studies that have evaluated sampling designs using different markers (Microsatellites, AFLPs, SNPs); empirical vs. simulated data; and varying the number of loci, individuals, and populations.

References	Dataset	Type of sampling	No. of populations	No. of individuals	No. of Loci	Principal conclusions
Miyamoto et al. (2008)	Microsatellite	Empirical	1	480	4	> 30 individuals increases the precision in *H*s Between 200 and 300 individuals increase the precision of allelic richness estimates.
Pruett and Winker (2008)	Microsatellite	Empirical	1	200	8	Precision in summary statistics is increased when > 20 individuals are genotyped.
González-Ramos et al. (2015)	Microsatellite	Empirical	2	64	15	Above 6 polymorphic markers are enough to adequately define the genetic structure between populations.
Peterman et al. (2016)	Microsatellite	Empirical	5	80	15	Increasing the number of loci does not change the mean summary statistics, but increases the precision across replicates. IBD patterns are sensitive to fewer loci genotyped.
Sánchez-Montes et al. (2017)	Microsatellite	Empirical	17–21 (different species)	547, 652, and 516	18, 16, and 15	> 20 individuals and between 50 and 80 individuals per population are needed to estimate *H*_*S*_ with precision, and allelic richness, respectively.
Rico (2017)	Microsatellite	Simulation	17 and 34 (different species)	5,000 and 3,000	20	Spatial sampling design (random, systemic, cluster) affect IBD patterns. Increasing loci, over individuals, increases the accuracy of IBD estimates.
Schwartz and McKelvey (2009)	Microsatellite	Simulation	1	10,000	15	Different sampling designs generate different *F*_*ST*_ estimates, and different *Structure* outputs.
Landguth et al. (2012)	Microsatellite	Simulation	1	1,000	25	Increasing the number of polymorphic loci increases the precision of patterns of isolation by resistance (IBR).
Oyler-McCance et al. (2013)	Microsatellite	Simulation	1	1,000	25	Increasing the number of polymorphic loci, individuals, and number of alleles increases the precision and the accurate estimation of patterns of (IBR).
Landguth and Schwartz (2014)	Microsatellite	Simulation	64	64	20	Increasing the number of populations (even if fewer individuals are sampled) increases the possibility of finding correct patterns of IBD.
Smith and Wang (2014)	Microsatellite	Simulation	3	100	100	Reducing the number of samples do not affect *Hs*, *F*_*ST*_ estimates, but reduces the power to detect accurate allelic richness.
Hale et al. (2012)	Microsatellite	Mixed (Simulation and empirical)	4	100	9, 5, 7, and 8	For four different species, sampling between 25 and 30 individuals are enough to estimate accurately *H*_*S*_ and *F*_*ST*_.
Dubois et al. (2017)	Microsatellite	Mixed (Simulation and empirical)	4	4 different taxa: 726, 408, 372, 384	16	Sex proportions do not affect summary statistics estimates. >20 individuals increase the precision of summary statistics. Empirical and simulated data show different patterns of deviation.
Sinclair and Hobbs (2009)	AFLPs	Empirical	6	159	59 and 117	>30 individuals per population needed to estimate accurately *F*_*ST*_.
Willing et al. (2012)	SNPs	Simulation	2	1,000	21,000	Fewer individuals are needed to accurately estimate *F*_*ST*_ for MPS datasets.
Fumagalli (2013)	SNPs	Simulation	1	1,000	20,000	Low individual sampling, with a high genome coverage underestimates the number of segregating sites, *H*_*S*_ estimates and genetic structure.
Nazareno et al. (2017)	SNPs	Empirical	2	70	3,500	Fewer individuals (8) but with a large number of SNPs (>1,000) increase the precision of *H*_*S*_ and *F*_*ST*_.
Flesch et al. (2018)	SNPs	Empirical	4	120	14,000	>25 individuals (with 10,000 SNPs) are needed to estimate accurate kinship indexes (10,000 SNPs), identifying as identical by descent alleles and *F*_*ST*_ values.
Puckett and Eggert (2016)	Mixed (SNPs and Microsatellite)	Empirical	34	Microsatellites dataset: 506 SNP dataset: 96	Microsatellite dataset: 15 SNP dataset: 1,000	1,000 SNPs are more precise than microsatellites for assigning birth areas, even if fewer individuals are sampled.

Sampling design has been studied widely. Nevertheless, the majority of the studies mentioned above (Table 1) were conducted mainly considering microsatellites, and thus focused on fewer loci and higher mutation rates than MPS data. In addition, studies centered on MPS markers were mostly based on bio-informatic simulations (Table 1). Among these, three studies have evaluated the effect of sampling design on estimates of genetic parameters using empirical and genomic data. Puckett and Eggert (2016) compared data for 15 microsatellite and 1,000 SNPs in *Ursus americanus* and found that the SNP dataset was more precise than the microsatellites in assigning the provenance of 96 individuals sampled across 34 populations. Nazareno et al. (2017) analyzed different sampling tests of *Amphirrhox longifolia* (Violaceae), ∼4,000 SNPs and 70 individuals distributed in two populations. They found that sampling over eight individuals per population and 1,000 SNPs did not increase the accuracy in the estimation of summary statistics. Flesch et al. (2018) analyzed four populations of rocky mountain bighorn sheep, 14,000 SNPs, and 120 individuals in total, finding that an accurate estimation of genetic parameters was achieved after sampling 25 individuals per population.

While the studies of Puckett and Eggert (2016), Nazareno et al. (2017), and Flesch et al. (2018) are without doubt informative and relevant, they were performed in most cases in relatively few populations (34, 2, and 4 populations, respectively) and were based in a relatively small number of individuals (96, 70, 120, respectively) or SNPs (1,000, ∼4,000, and ∼14,000, respectively). More importantly, these studies did not test the effect of sampling design on the detection of outlier SNPs using empirical data.

In this study we aimed at testing the effect of sampling design to assess the potential biases and errors in estimates of population genomics parameters, in patterns of isolation, and in the detection of outlier SNPs while using empirical datasets. For this, we compared two simulated data sets, two large genomic datasets (33,454 SNPs, 646 individuals, and 49 populations obtained with the MaizeSNP50 Genotyping BeadChip; and 9,735 SNPs, and individuals pooled from 47 populations obtained with the DArTseqTM data), and one microsatellite dataset (22 microsatellite loci, 527 individuals, and 29 populations) of Mexican wild maize populations (*Zea mays* ssp. *mexicana* and *Zea mays* ssp. *parviglumis*) to explore the effects of sampling design in the estimation of population genomics parameters (*H*s, *F*_*IS*_, *F*_*ST*_), landscape genetics (tests of isolation by distance and environment), test of centrality (association between genetic diversity and the distance from the center of the geographic or niche distribution; Eckert et al., 2008; Lira-Noriega and Manthey, 2014; Aguirre-Liguori et al., 2017), and estimation of candidate SNPs (outlier SNP detection tests).

In particular, we compared the effect of (1) using MPS vs. microsatellites markers; (2) using individual data with known ascertainment bias (MaizeSNP50 Genotyping BeadChip) vs. pooled non-ascertained biased data (DArTseqTM data); (3) varying the number of sampled loci (genomic datasets: 100, 1,000, 5,000, 15,000; microsatellite datasets: 5, 10, 15); (4) varying the number of sampled individuals per population (3, 6, and 9 individuals); (5) changing the number of sampled populations (5, 10, 20, 30, 40 populations); and (6) testing the effect of the number of sampled populations in the detection of outlier SNPs.

## Materials and Methods

### Studied Taxon

Mexican wild maize, or teosintes, are divided into two main subspecies, the lowland subspecies *Zea mays* ssp. *parviglumis* (hereafter *parviglumis*) and the highland subspecies *Zea mays* ssp. *mexicana* (hereafter *mexicana*) (Aguirre-Liguori et al., 2016). Demographic studies suggest that *mexicana* was originated from *parviglumis* between 20,000 and 60,000 years ago and that divergence occurred in the presence of gene flow (Aguirre-Liguori et al., 2019a). Consequently, the Mexican wild teosintes fit a model of hierarchical gene flow, with higher gene flow occurring within subspecies. Given the close relatedness of teosintes to maize, different genomic resources are available (Hufford et al., 2012; Aguirre-Liguori et al., 2016) and several studies have analyzed their population genomics (van Heerwaarden et al., 2011; Hufford et al., 2013; Pyhäjärvi et al., 2013; Aguirre-Liguori et al., 2017, 2019a, b; Fustier et al., 2017, 2019; Moreno-Letelier et al., 2020). Briefly, genomic studies suggest that teosintes have high genetic diversity, show patterns of isolation by distance and environment, and show strong patterns of local adaptation (Pyhäjärvi et al., 2013; Aguirre-Liguori et al., 2017, 2019a, b; Fustier et al., 2017, 2019). The vast genomic resources and biological knowledge makes teosintes an ideal system to study the importance of sampling design in analyses of genetic diversity, isolation patterns, and identification of candidate SNPs.

### Datasets

#### Simulated Datasets

The majority of tests that have analyzed the effect of sampling design on the estimations of summary statistics using genomic information have been performed with simulated data (Table 1). The advantage of using simulated data is that it allows for modeling an evolutionary process based on known demographic parameters. Here we simulated two large genomic datasets to analyze the effect of sampling design on the estimations of summary statistics and then compared the results with two empirical genomic datasets and one microsatellite dataset.

We used Fastsimcoal 2 (Excoffier and Foll, 2011; Excoffier et al., 2013) to simulate two demographic models consisting of 50 populations divided into two genetic clusters fitting a model of hierarchical structure (i.e., two subspecies of teosintes). Populations belonging to the old genetic cluster (i.e., *parviglumis*) coalesced with their common ancestor approximately 140,000 generations ago (Ross-Ibarra et al., 2009). Populations belonging to the young genetic cluster (i.e., *mexicana*) coalesced with their common ancestor approximately 20,000 generations ago (Aguirre-Liguori et al., 2019a). We set the time of divergence between the two genetic clusters at ∼20,000 generations ago (Aguirre-Liguori et al., 2017).

The effective population size of the old genetic cluster was set to ∼5,000 individuals and was 1.5 times higher than the young genetic cluster. For the first model (the hierarchical model with high gene flow), migration between populations belonging to the same genetic clusters were set at a 0.001 probability of a gene moving from one population to the other back in time. The migration between populations belonging to different genetic clusters were 10 times smaller (0.0001). For the second model (the hierarchical model with low gene flow), gene flow did not occur between populations belonging to different genetic clusters.

To incorporate variation in the demographic parameters across populations, we used the *norm* function in R to create a normal distribution for each demographic parameter (*N_*e*_, m, T*, and inbreeding index) with the mean values detailed above. Next, for each population we sampled a random value for each parameter.

We created the fastsimcoal inputs using the *fscWrite* function of the *strataG* package of R. For each model we used the command line *fsc26 –i input –n 1 –g –I* to simulate 30,000 SNPs (with an infinite site model) and 15 diploid individuals per population. Finally, we used *strataG* (Archer et al., 2016) and the *adegenet* (Jombart, 2008) pacakage of R 3.6.1 (R Core Team, 2019) to create for each simulated dataset a *genind* and a *hierfstat* input object for further analyses.

#### Empirical Datasets

For the empirical datasets, we combined the MaizeSNP50 Genotyping BeadChip data published by Pyhäjärvi et al. (2013) and Aguirre-Liguori et al. (2017) to obtain a total dataset consisting of 49 populations, 24 belonging to *mexicana* and 25 to *parviglumis* (Supplementary Figure S1), including between 12 and 15 individuals per population, and 33,454 SNPs. Since the MaizeSNP50 Genotyping BeadChip was designed to maximize variation in maize, it has ascertainment bias (Albrechtsen et al., 2010). Therefore, this dataset is expected to include SNPs that are in high frequency across distant teosintes populations and thus might overestimate genetic diversity and underestimate genetic differentiation.

We also downloaded the DArTseq data from Aguirre-Liguori et al. (2019a), which are composed of pooled DNA of 47 populations (∼12 individuals per population), 21 belonging to *parviglumis* and 26 to *mexicana* (Supplementary Figure S1), and 9,735 SNPs. The DArTseq dataset was obtained by initially cutting the DNA using restriction enzymes (Sansaloni et al., 2011; Ren et al., 2015) and has lower ascertainment bias (see Aguirre-Liguori et al., 2019a). This dataset has a frequency spectrum with lower bias than the 50K dataset and is expected to generate more robust demographic inferences (Albrechtsen et al., 2010). We called the BeadChip and the DArTseq datasets the 50K and DTS datasets, respectively.

To be able to compare deviations obtained from MPS and microsatellite markers (Table 1), we also used the microsatellite dataset from Gasca-Pineda et al. (2020), which includes 527 individuals distributed across 29 populations, 14 belonging to *parviglumis* and 15 to *mexicana* (Supplementary Figure S1). This microsatellite dataset consists of 22 loci and 355 alleles.

For each population and dataset, we downloaded the longitude and latitude at which they grow (Supporting Information in Aguirre-Liguori et al., 2017, 2019a; Gasca-Pineda et al., 2020). We also obtained the score of the first principal component (PC1) describing temperature for each population. The environmental data were obtained from 19 bioclimatic variables downloaded from WorldClim at a 30°arc Resolution, and the PCA was performed using the prcomp function in R across all variables and populations.

These three datasets share many populations (Supplementary Figure S1). The microsatellite dataset is a subsample of the 50K dataset and therefore shares all populations with the 50K dataset. The 50K and DTS datasets shared 29 populations. Also, the three datasets are distributed along the entire geographic and environmental distribution of teosintes (Hufford et al., 2012; Pyhäjärvi et al., 2013; Aguirre-Liguori et al., 2017, 2019a). They are composed of many individuals per population (between 9 and 26 individuals per population) and contain a large number of SNPs or microsatellite markers, distributed along the 10 chromosomes of teosinte. Importantly, the 50K and DTS datasets are the largest genomic datasets based on population sampling (not accessions) that have been developed so far in teosintes. Therefore, we considered these datasets (and the microsatellite dataset) as the samples representing the most accurate data (i.e., the “real” data for the purpose of this work) and estimated the deviations in the estimations of summary statistics, landscape genetics, and tests for local adaptation, generated by sampling a different number of loci, the number of individuals, and the number of populations.

We used *adegenet* and *hierfstat* (Goudet, 2005) packages of R to generate *genind*, *genpop*, and *hierfstat* objects to manipulate the data. All these objects are indexable, and therefore allow subsampling random individuals, SNPs, microsatellite markers, subspecies, and/or populations.

For all subsamplings we combined the *mexicana* and *parviglumis* populations. However, complex demographic scenarios can bias the estimations of divergence between populations when only few populations are sampled (Chikhi et al., 2010; Heller et al., 2013; Robinson et al., 2014). For instance, hierarchical structure increases the *F*_*ST*_ between populations belonging to different genetic groups (Slatkin and Voelm, 1991) and reduced sampling can bias estimations of divergence if more populations are sampled within one genetic cluster than between genetic clusters. Alternatively, incomplete lineage sorting can underestimate the amount of divergence between populations belonging to different genetic clusters (Lack et al., 2010; Orozco-Terwengel et al., 2011; Jones, 2019). To test the effect of sampling bias associated with complex demographic structures, we also tested the effect of sampling design by analyzing each subspecies separately. Since the patterns were similar between the entire datasets and the subspecies datasets, for simplicity we present the results of the combined datasets and show results of each subspecies as Supplementary Information.

### Estimation of Population Genetics Parameters

For each simulated dataset, the entire genomic datasets, and each subsampling within dataset (see below for descriptions of the subsamplings), we used the *basic.stats* function of the *hierfstat* package in R to calculate the sample *H*_*S*_ and *F*_*IS*_ and *F*_*ST*_. For each summary statistic, we obtained the mean value across loci for each population.

For the empirical datasets, we also used environmental data to analyze landscape genetic associations. For the genomic datasets, we used the *BEDASSLE* package (Bradburd et al., 2013) in R to calculate the pairwise *F*_*ST*_ between populations (Weir and Hill, 2002). For the microsatellite datasets, we used the *pairwise.fst* function of the *hierfstat* package in R to calculate Nei’s pairwise *F*_*ST*_ between populations.

We tested patterns of isolation by distance (IBD) and IBE using multiple regressions of distance matrices (*MRM* function from the *ecodist* package; Goslee and Urban, 2007) to test the association between pairwise genetic distance (*F*_*ST*_) as a response variable and the environmental and geographic distances as predictive variables. We performed 1,000 permutations in each test. The advantage of MRM tests is that they allow for simultaneous testing in both the environmental and geographic distances, and determine the relative contribution of each variable (Lichstein, 2007).

Finally, we tested the central abundance hypothesis (CAH), which suggests that genetic diversity should reduce as a function of the distance from the geographic or niche centroid (Eckert et al., 2008; Martínez-Meyer et al., 2012; Lira-Noriega and Manthey, 2014). For the CAH tests, we used simple linear regressions (*lm* function in R) to test the association between *H*s as the response variable and the distance to the niche and geographic centroids as independent variables (which were estimated as the Euclidian distances from the geographic and niche centroids; for more details of the methods see Aguirre-Liguori et al., 2017).

### Sampling Designs

First we analyzed the effect of sampling design with the estimation of *H*s, *F*_*IS*_, and *F*_*ST*_ using the simulated dataset. Since we controlled the demographic parameters of the simulations, we were able to generate an expectation of how sampling design would affect the estimation of summary statistics. Next, we used the empirical dataset to validate the simulated results.

#### Subsampling of the Number of Loci and the Number of Individuals per Population

We tested the effect of sampling a different number of SNPs or microsatellite markers per population. We used R custom scripts (available as Supporting Information- function num_locs) to extract data from the entire empirical and genomic datasets: for the DTS dataset 100 (∼1%), 1,000 (∼10%), and 5,000 (∼51%) random SNPs; for the 50K dataset and the simulated datasets100 (∼0.3%), 1,000 (∼3%), or 15,000 (∼45%) random SNPs; and for the microsatellite dataset 5 (∼22%), 10 (∼45%), and 15 (∼68%) random markers. For the simulated, the 50K, and the microsatellite datasets, we also tested the effect of sampling different estimates of individuals per populations. We extracted randomly for each population 3, 6, and 9 individuals [available as Supporting Information- function num_inds()]. This was not performed on the DTS dataset, because it was based on pooled DNA.

For the number of SNPs, the number of microsatellite markers, and the number of individuals per population, we re-sampled randomly and without replacement each set 1,000 times, we generated *genind*/*hierfstat*/*BEDASSLE* input objects and estimated the summary statistics described above (*H*s, *F*_*IS*_, *F*_*ST*_, IBE, IBD, CAH associations). For each parameter and each replicate we obtained the mean summary statistic across populations and generated a distribution based on 1,000 summaries corresponding to each subsampling.

#### Subsampling the Number of Populations

To test the effect of the number of populations in the estimation of the parameters described above (*H*s, *F*_*IS*_, *F*_*ST*_, IBE, IBD, CAH associations), we performed random sampling designs. For the simulated and the genomic datasets, we sampled 5 (∼10%), 10 (∼20%), 20 (∼40%), 30 (∼61%), and 40 (∼81%) random populations from the 49 (50K) and 47 (DTS) populations described above (Supplementary Figure S1). For the microsatellite dataset, we sampled 5 (∼17%), 10 (∼34%), and 20 (∼69%) random populations from the 29 populations described above (Supplementary Figure S1). Again, we generated 1,000 subsamples without replacement [available as Supporting Information- function num_pops ()], and for each replicate we generated *genind*/*genpop*/*hierfstat*/*BEDASSLE* inputs and in each case, we calculated the estimates described above, and for summary statistics we estimated the mean across populations.

#### Tradeoff Between Number of Individuals and Populations

To test the tradeoff between the number of individuals and number of populations, for the 50K dataset we also tested three sets of sampling designs changing the number of individuals sampled per population, going from fewer individuals in many populations to many individuals sampled in fewer populations. For three scenarios (3 individuals and 49 populations; 6 individuals and 24 populations; 9 individuals and 10 populations) we generated 1,000 subsamples and estimated the parameters described above.

#### Comparison Between Samplings and Between Datasets

For each subsampling, we compared qualitatively the simulations to the “real” dataset, to determine the deviations generated by different sampling designs. To be able to compare between different datasets (including the simulated and empirical datasets), we also compared the magnitude of the deviation between sampling designs and between datasets using the relative error between each subsampling and the estimated “real” summary statistics described above. The relative error was calculated as *(X_*est*_ – X_*sim*_)/X_*est*_*, where *X*_*est*_ is the summary statistic estimated for the “real data” set and *X*_*sim*_ is the summary statistic estimated for a given subsampling.

### Test for Local Adaptation

Detecting outlier SNPs is challenging, since high genetic structure can inflate false positives (Schoville et al., 2012; De Mita et al., 2013; Tiffin and Ross-Ibarra, 2014). We tested the effect of varying the number of populations in detecting outlier loci. For this, we subsampled without replacement 5, 10, 20, and 30 random populations from the entire 50K dataset (49 populations and between 12 and 15 individuals per population, see Aguirre-Liguori et al., 2017 for more details). Since outlier analyses are time-consuming, we only generated 10 replicates of each sampling design, and we subsampled 10,000 SNPs from the 50K dataset. We also ran the analysis 10 times with the 49 populations to have a comparable number of replicates. We chose 10,000 SNPs to reduce computing time and because our results (see below) show that over 1,000 SNPs are enough to identify adequately the genetic structure between populations, and therefore reduce false positives.

For each sample, we used *Bayescenv* (De Villemereuil and Gaggiotti, 2015) to identify outlier SNPs associated to PC1 (as in Aguirre-Liguori et al., 2017, 2019a). *Bayescenv* decomposes *F*_*ST*_ based on a signal shared between all loci (β), a signal specific to each locus (α), and the association of the SNP with the environmental variable tested (γ). We used default parameters to run the analyses and we defined outlier SNPs as those that had *q-val* < 0.05, which is a conservative approximation to detect outlier loci (De Villemereuil and Gaggiotti, 2015). For each replicate of each sampling design, we recorded the highest *F*_*ST*_ value for a SNP and the number of SNPs that had *q-val* < 0.05.

We used the entire dataset to identify outlier SNPs. We considered this dataset as presenting the “real outlier SNPs” representing the local adaptation to all environmental conditions in which teosintes grow. We tested whether different sampling designs based on a different number of populations sampled would identify a subset of the outlier SNPs detected for the entire dataset. We used the *intersect* function in R to detect the SNPs that were considered as “outlier” for all replicates in each sampling design. We also used the *venn* function of the *gplots* package in R to identify SNPs that were the candidate (q-val) for all sampling designs (5, 10, 20, 30, and 49 populations) and the 10 replicates.

## Results

### Summary Statistics for the Entire Datasets

#### Simulated Datasets

We generated two simulated datasets with hierarchical structures, but with different levels of gene flow between populations belonging to different genetic clusters.

For the two models, we found that estimated genetic diversity was high and the fixation index low (Table 2 and Supplementary Figure S2), as it has been found in teosintes. As expected, we found that the hierarchical model with high gene flow had a lower mean *F*_*ST*_ than the hierarchical model with low gene flow (Table 2 and Supplementary Figure S2). Importantly, we found large variance between populations for *H*s and *F*_*IS*_, which is similar to what has been observed in teosintes (Aguirre-Liguori et al., 2017). The values of *F*_*ST*_ were close to 0, and for many replicates we found negative values approximate to 0 (see Supplementary Figure S2). Negative *F*_*ST*_ values occur when sample size corrections are used, and are usually considered to be 0. However, to be able to compare the relative error associated to sampling design, we recorded the *F*_*ST*_ estimated from *hierfstat*.

**TABLE 2:** Summary statistics estimated for the DTS, 50K, and microsatellite datasets of Mexican wild maize.

Mean estimate	Hierarchical high flow	Hierarchical low flow	DTS	50K	Microsatellite
*H*_*S*_	0.26 (0.05)	0.32 (0.03)	0.130 (0.05)	0.225 (0.04)	0.691
*F*_*IS*_	0.02 (0.18)	0.01 (0.18)		0.069 (0.04)	0.182
*F*_*ST*_			0.393	0.246	0.106
MRM: geographic (β)			0.027	0.025	0.013
MRM: environmental (β)			0.011	0.011	0.004
CAH: geographic (β)			−0.014	−0.014	−0.041
CAH: environmental (β)			−0.006	−0.008	−0.031

*The numbers in parenthesis correspond to standard deviation of the mean values. For the mean and maximum and minimum values across 1,000 replicates of each sampling designs (see Supplementary Table S1).*

In brief, we consider that the simulated datasets were adequate datasets to generate expectations of how sampling designs would affect the estimation of summary statistics.

#### Empirical Datasets

We considered the entire datasets (50K, DTS, and microsatellite) as those revealing the “real” or most accurate patterns of genetic diversity across teosinte populations. Table 2 shows the mean *H*_*S*_, *F*_*IS*_, and *F*_*ST*_ across populations, patterns of isolation by distance and environment, and the test of central abundance, estimated for the DTS, 50K, and microsatellite datasets (the distribution across different sampling designs are found in Supplementary Table S1).

**FIGURE 1:**
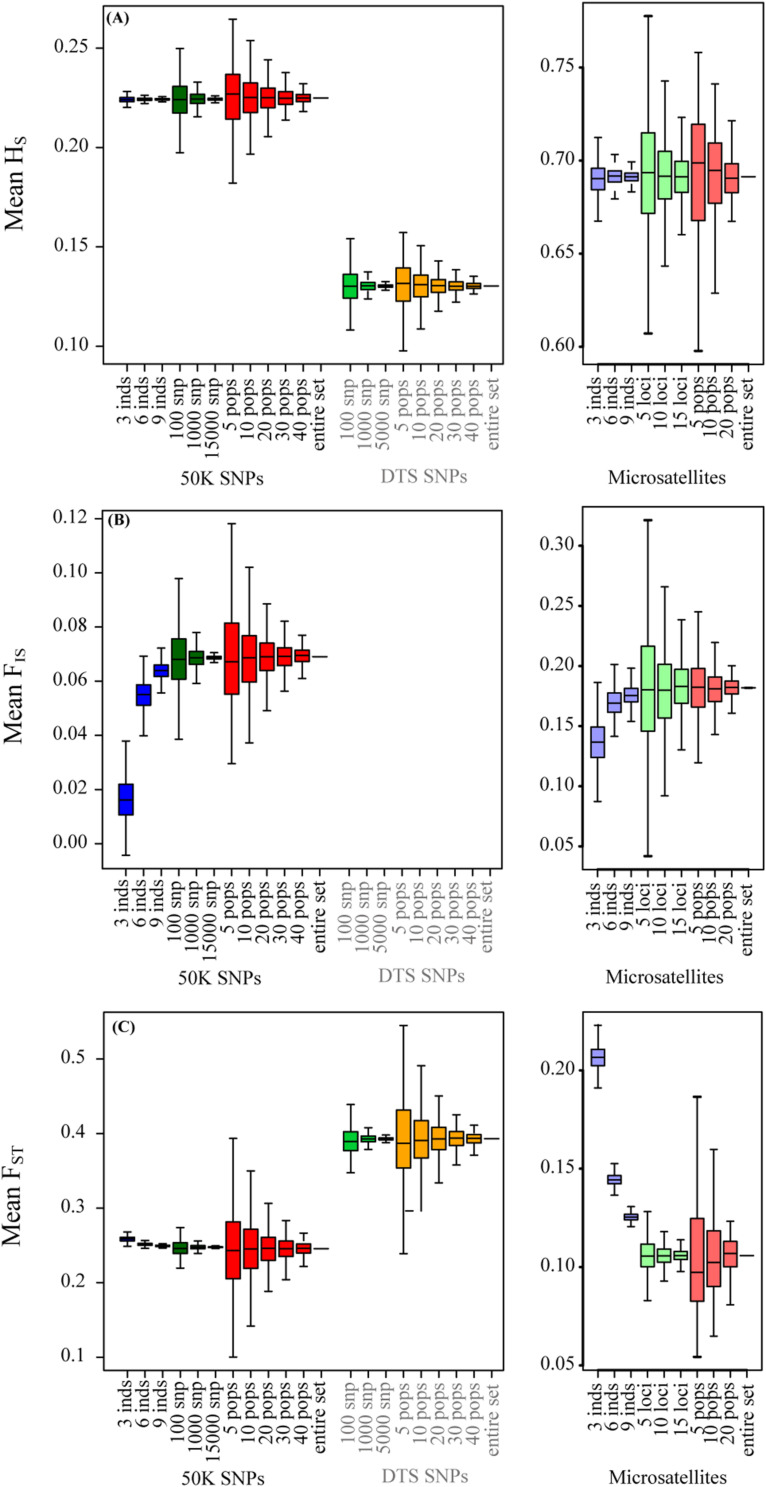
The effect of sampling designs on the estimation of summary statistics for genomic (left panels) and microsatellite (right panels) datasets: **(A)** *H*_*S*_; **(B)** *F*_*IS*_; **(C)** *F*_*ST*_. Boxplots show the distribution of mean summaries estimated for 1,000 replicate simulations varying the number of individuals, number of SNPs, and number of populations sampled. *F*_*IS*_ was not possible to obtain for the DTS dataset because it is based on pooled data.

We found striking differences among the datasets for the estimated mean across populations of *H*s, *F*_*ST*_, and *F*_*IS*_ (Figure 1 and Table 2). We detected that the DTS dataset presents low mean genetic diversity across populations (*H*s = 0.13), the 50K intermediate values (*H*s = 0.225), and the microsatellite data high values (*H*s = 0.69). In parallel fashion, we found that DTS shows the highest mean genetic structure across populations (*F*_*ST*_ = 0.393), followed by the 50K dataset (*F*_*ST*_ = 0.246), and finally the microsatellite dataset (*F*_*ST*_ = 0.11). We were not able to calculate *F*_*IS*_ for the DTS dataset (as they were derived from pooled DNA), but we also found differences between the estimated mean using the 50K (*F*_*IS*_ = 0.065) and microsatellite datasets (*F*_*IS*_ = 0.19).

**FIGURE 2:**
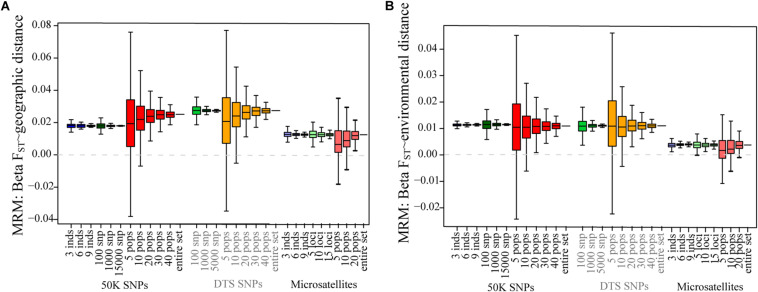
The effect of sampling designs on the analysis of patterns of isolation for genomic and microsatellite datasets: **(A)** IBD-MRM test; **(B)** IBE-MRM test. Boxplots show the distribution of associations estimated for 1,000 simulations varying the number of individuals, number of SNPs, and number of sampled populations. The dotted gray line shows the 0 value.

**FIGURE 3:**
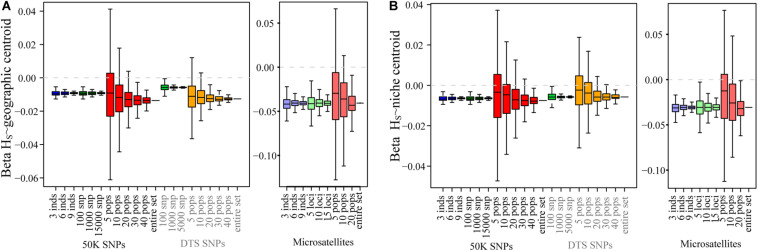
The effect of sampling designs on the estimation of the central abundance hypothesis for genomic and microsatellite datasets: **(A)** the association between distance to the geographic centroid and *H*s; **(B)** the association between distance to the niche centroid and *H*s. Boxplots show the distribution of associations estimated for 1,000 simulations varying the number of individuals, number of SNPs, and number of sampled populations. The dotted gray line shows the 0 value.

In contrast to the summary statistics, for the three datasets we found similar patterns of IBD and IBE (Figure 2), based on the MRM tests (Figure 2 and Table 2). For the three datasets, we observed that patterns of IBD and IBE were positive, indicating that there is isolation by distance or by environment. Finally, for the three experimental datasets we observed negative associations between genetic diversity and the distance to the geographic and niche centroids (Figure 3 and Table 2), indicating that as populations grow further away from the center of their geographic distribution or the optimum ecological conditions, their genetic diversity is lower.

### Varying the Number of Sampled Individuals

As mentioned above, this test was performed with the simulated datasets, the 50K, and the microsatellite datasets, since they were based on individual samples. The DTS dataset was generated from pooled DNA and therefore individual genotypes were not known.

For the two simulated datasets, we found that subsampling fewer individuals increased the variance and relative error in the estimation of *H*s, *F*_*IS*_, and *F*_*ST*_ across 1,000 replicates (Supplementary Figure S2). Importantly, for *F*_*IS*_ estimations we found that when fewer individuals were sampled, the mean value across the 1,000 replicates was lower than the complete dataset, indicating that this summary statistic was the most sensitive to the number of individuals sampled.

These patterns were similar for the empirical 50K and microsatellite datasets. Briefly, we found that sampling fewer individuals did not strongly affect the estimates of *H*s (Figure 1A and Supplementary Figure S3). Also, in accordance to the simulated data, we found that changing the number of sampled individuals generated strong deviations and a higher relative error for the estimation of *F*_*IS*_ for the 50K and microsatellite datasets (Figure 1B and Supplementary Figures S3, S6).

In contrast to the simulated datasets, we found that sampling a different number of individuals generated moderate deviations for estimates of *F*_*ST*_ for the 50K dataset (Figure 1C and Supplementary Figures S3, S6) and large deviations for the estimates of *F*_*ST*_ for the microsatellite dataset (Figure 1C and Supplementary Figures S3, S6). We found that the relative error was higher for *F*_*IS*_ estimations when using the 50K dataset, but higher for microsatellites when estimating *F*_*ST*_ (Supplementary Figure S6). Importantly, in both datasets we found that sampling fewer individuals reduced the *F*_*IS*_ estimates and increased the *F*_*ST*_ estimates (Figures 1B,C).

For the empirical datasets, we also estimated the effect of sampling a different number of individuals in different landscape genetic tests. Even if we found increased variance in the estimation of *F*_*ST*_, it was interesting to note that we did not find strong deviations for summary statistics describing patterns of isolation by distance and environment (Figure 3 and Supplementary Figure S4). Finally, in accordance to low variance in the estimation of *Hs*, we found low biases in the associations between genetic diversity and ecological variables (Figure 3 and Supplementary Figure S5). The ranges of the maximum and minimum values were close to the “real” estimates for the 50K dataset.

It is interesting to note that *F*_*IS*_ was the only summary statistic that was very sensitive to the number of individuals sampled for the simulated datasets, and the empirical 50K and microsatellite datasets. Since this pattern is shared between the simulated and the empirical datasets, the differences are not associated with increased missing data or null alleles. To identify what could be generating these differences, we analyzed the *F*_*IS*_ values for all loci across populations. We found that fewer individuals sampled generated larger “NaN (Not a Number)” values across loci. This is the result of loci appearing as fixed since a larger number of individuals are need to sample low frequency alleles. We correlated the mean *F*_*IS*_ across populations and the number of “NaN” values estimated per loci per population and found that the reduction in *F*_*IS*_ correlated with an increased number “NaN” obtained when fewer individuals were sampled (Supplementary Figure S7).

### Varying the Number of Sampled Loci

Next, we evaluated the effect of sampling a different number of loci in the estimation of summary statistics. For the two simulated datasets, we found that sampling fewer loci slightly increased the relative error and the variance in the estimation of the summary statistics across replicates (Supplementary Figure S2). Interestingly, the variance and relative error was stronger for *H*s estimations when 100 loci were sampled than for the other summary statistics (Supplementary Figure S2). Sampling 1,000 and 15,000 random SNPs did not generate strong differences.

Sampling a different number of loci for the three empirical datasets showed similar patterns to the simulated datasets. In all cases, sampling fewer loci increased slightly the variance and the relative error across replicates for all estimates (Figures 1–3 and Supplementary Figures S3–S6). Importantly, we found that the variance and relative error across replicates was higher for the microsatellite dataset (especially when sampling five microsatellites, Supplementary Figure S6), followed by the DTS dataset, and finally when sampling each subspecies using the 50K dataset (Supplementary Figures S3–S5).

Even though decreasing the number of loci increased the variance, it was interesting to note that estimated distributions fell close to the estimates for “real” datasets (Supplementary Table S1). For *H*_*S*_ and *F*_*IS*_, sampling fewer microsatellite loci produced an important increase in the variance across replicates.

We also tested the effect of sampling a different number of loci in the estimation of landscape genetic statistics. In these tests we only found deviations with respect to the “real” datasets for the association between *H*_*S*_ and the geographic centroid, where we found that sampling fewer DTS and 50K SNPs reduced the association (β got closer to 0).

Importantly, sampling 1,000 or 15,000 of the 50K SNPs; 1,000 or 5,000 of the DTS SNPs; and 10 or 15 loci of the microsatellite dataset generated similar summary statistics and reduced the relative error in the estimation of the parameter (Supplementary Figure S6).

### Varying the Number of Sampled Populations

Finally, we tested the effect of sampling a different number of populations in the estimation of summary statistics. For the simulated datasets, we found that sampling fewer populations increased the variance and relative error across the three summary statistics. For all summary statistics we found that the mean value across the 1,000 replicates of the different number of populations sampled were similar to the entire dataset, except for the distribution of *F*_*ST*_ in the hierarchical model with low gene flow where we found a lower mean value as fewer populations were sampled (Supplementary Figure S2).

For the empirical datasets, varying the number of populations generated similar mean values to those found for the “real” datasets. Also, for the three datasets, sampling a small number (5 and 10) of populations generated deviation and importantly increased the relative error compared to the real estimated values for all summary statistics and in particular for patterns of isolation by distance and environments and patterns associated with the tests of centrality (Figures 2, 3).

The variance and relative error across datasets dropped after approximately 30 populations for the genomic datasets (see ranges in Supplementary Table S1 and Supplementary Figures S5, S6), but remained high for the microsatellite dataset. The relative error was in general higher for samplings using the DTS dataset, except for patterns associated with the test of centrality, for which the 50K dataset presented a higher error (Supplementary Figure S6). Also we found that when sampling fewer populations, the microsatellite dataset presented a lower relative error than the 50K and DTS datasets (Supplementary Figure S6).

Importantly, sampling fewer populations generated a high variance and higher relative error in the estimation of all parameters, except for *F*_*IS*_ and *F*_*ST*_ for the microsatellite dataset (Figures 1B,D) and *F*_*IS*_ estimates for the 50K dataset (Figure 1 and Supplementary Figures S3–S6). In these cases, the deviations across replicates when sampling fewer populations was lower than the variance generated by sampling a different number of individuals (Figure 1 and Supplementary Figure S6).

For patterns of IBD and IBE, we also found that sampling fewer than 10 populations generated in some replicates incorrect association estimates. The real value showed positive associations, isolation by distance or environment (Table 2), but for 5 and 10 sampled populations we found that up to 18 and 7% of sample replicates generated negative associations, respectively (Supplementary Table S2; see changes in signs in Supplementary Table S1). For tests of association between *H*_*S*_ and ecological variables, this was even more sensitive for the genomic datasets, since we found up to 4.6% of positive estimates (when the entire dataset was negative) for 30 populations when testing association between *H*s and the distance to the niche centroid. However, we found that the DTS was less sensitive to deviation for associations between ecological variables and summary statistics (Figure 3).

Finally, we found that the microsatellite dataset was more sensitive for isolation by distance and environment patterns (we found up to 30% of samples showed opposing patterns to the entire dataset when sampling 5 populations; Supplementary Table S2), and less sensitive for tests of CAH (we found up to 1.8% of opposing results when sampling 20 populations; Supplementary Table S2).

### Tradeoff Between Number of Individuals and Number of Populations

**FIGURE 4:**
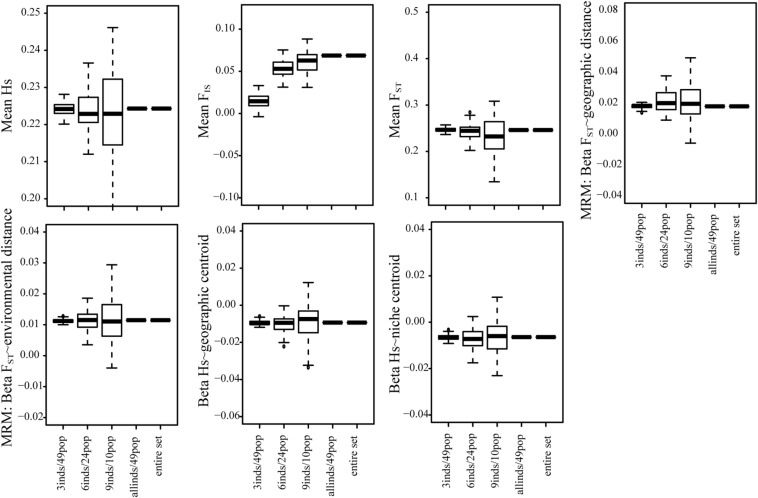
The tradeoff between the number of individuals and the number of populations sampled for all summary statistics using the 50K dataset. We tested the effect of sampling more individuals in fewer populations and fewer individuals in many populations.

For the 50K dataset, we also contrasted the effect of sampling fewer individuals in many populations or many individuals in fewer populations (3 individuals/49 populations, 6 individuals in 24 populations, and 9 individuals in 10 populations). For all summary statistics, except *F*_*IS*_, we found that sampling more populations but fewer individuals generated more accurate results and lower biases (Figure 4 and Supplementary Figure S8).

### Varying the Number of Populations in the Identification of Candidate SNPs

**FIGURE 5:**
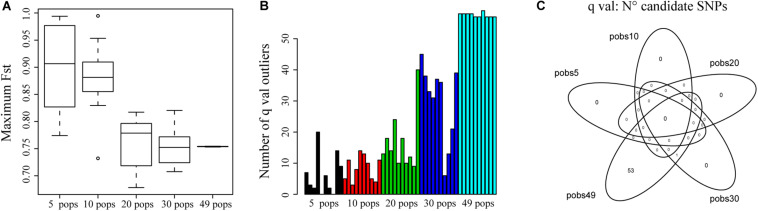
The effect of sampling a different number of populations on the identification of outlier SNPs. **(A)** Distribution of the highest *F*_*ST*_ identified for a locus across simulations. **(B)** Number of outlier SNPs (*q-val*) identified for each replicate for a different number of sampled populations. **(C)** The Venn diagram shows the number of shared SNPs identified across replicates and the number of populations sampled using q-val to identify outliers.

Figure 5A shows that for 5 and 10 sampled populations, the maximum *F*_*ST*_ for a locus found by *bayescenv* across replicates was higher than for the rest of the sampling designs. We also tested the number of candidate SNPs across replicates. We found more candidate SNPs when sampling a higher number of populations (Figure 5B).

We also evaluated how many shared outlier SNPs were identified by all replicates and sampling designs. Interestingly, we only found 1 SNP that was identified for the 10 replicates of the 30 and 49 populations sampling designs (Figure 5C). The lack of shared SNPs could be explained by the nature of *Bayescenv*, that identifies genome by environment associations. If the populations that were sampled in each test have different ecological settings, we would not expect to find the same outlier SNPs. Therefore, we also analyzed each replicate independently to identify how many outlier SNPs were shared with the 49 sampling designs (Supplementary Table S3). For replicates of 5 and 10 populations, less than 10% of SNPs were shared with the SNPs identified for 49 populations. For 20 populations, one replicate identified 46% of shared SNPs with 49 populations; and for 30 populations 4 replicates identified > 51% of shared outlier SNPs (Supplementary Table S3).

## Discussion

It remains challenging for many researchers to generate large genomic samples, posing a tradeoff between the information obtained with MPS and the number of populations sampled (Meirmans, 2015). Here we used two simulated datasets to estimate the effect of sampling size on the estimation of summary statistics. To confirm the effect on simulated results, we used empirical genomic datasets and a microsatellite dataset obtained for a large sample of wild maize, the teosintes (*Zea maiz* ssp. *parviglumis* and *Zea mays* ssp. *mexicana*), to test if the deviations generated by different sampling designs, while estimating classic population genomics and landscape genomics estimates. Depending on the objectives, and the amount of data that can be produced using genomic platforms, we propose some suggestions for sampling designs that could be considered according to our results (Table 3). It is important to consider that these recommendations might be more reliable for species with life history traits similar to Mexican wild maize, and caution should be taken since life history can have an important effect in summary statistics (Hamrick and Godt, 1996; Nybom, 2004).

**TABLE 3:** Recommendations for sampling designs depending on study objectives.

Estimate	Number of individuals	Number of loci	Number of populations	Considerations
*H*_*S*_	Not sensitive (> 6 individuals)	Sensitive (>1,000 SNP loci; > 15 microsatellite loci)	Sensitive (> 20 populations)	Increase the number of loci and populations. Genomic dataset is less sensitive than microsatellite dataset.
*F*_*IS*_	Very sensitive (>9 individuals)	Sensitive (>1,000 SNP loci; > 15 microsatellite loci)	Sensitive (> 20 populations)	Increase the number of individuals over loci and populations. If fewer populations are available, increase the number of individuals in those populations.
*F*_*ST*_	Microsatellite dataset was very sensitive (> 20 individuals). 50K dataset: Not sensitive (>9 individuals)	Not very sensitive (>1,000 SNPs; > 15 loci)	Sensitive (> 20 populations)	Increase the number of populations over the number of SNPs or individuals.
IBD and IBE MRM tests	Not sensitive (> 3 individuals)	Not sensitive (>1,000 SNPs, > 15 loci)	Very sensitive (>20 populations)	Sample as many populations as possible even if fewer individuals or loci are sampled.
CAH tests	Not very sensitive (> 3 individuals for genomic datasets; > 6 individuals for microsatellite datasets)	Sensitive depending on the dataset (>1,000 DTS SNPs, > 100 50K SNPs, > 15 microsatellite loci)	Very sensitive (> 30 populations)	Increasing the number of populations is more important than increasing the number of loci or individuals. Microsatellites are more sensitive than genomic datasets to the number of loci and individuals, although less sensitive to the number of populations sampled.
Tests of selection using bayescenv	Not tested	Sensitive (as many as possible)	Very sensitive (>30–40 populations)	As many SNPs as possible are needed to differentiate outlier loci, also to increase the probability of finding loci within selective regions. Increase as much as possible the number of populations, covering the largest geographic and environmental distribution. A possibility is to use pooled-sample DNA.

*The numbers within parenthesis indicate how many individuals, populations, or loci should be sampled for different objectives according to our simulations.*

### Comparing Datasets

We used Fastsimcoal 2 as a tool to simulate large samplings and complex demographic scenarios similar to teosintes. Except for *F*_*ST*_ estimations, we found that the values of genetic diversity and variance between populations (Table 2) were similar between the simulated and empirical dataset.

For the empirical datasets, we found that the most evident differences between datasets were associated to *H*_*S*_, *F*_*ST*_, and *F*_*IS*_ estimates. These differences are expected, given the properties of each dataset. First, we found that the microsatellites had the highest *H*_*S*_ and lowest *F*_*ST*_, which is a well-known pattern, and can be explained by their large number of alleles and mutation rates (Ellegren, 2004). Second, the 50K dataset had higher genetic diversity and lower *F*_*ST*_ compared to the DTS dataset. This is concordant with the design of the 50K dataset to detect highly polymorphic SNPs in maize, and therefore has ascertainment bias (Albrechtsen et al., 2010). In contrast, the DTS dataset was generated using restriction enzymes (similar to GBS and other reduced representation methods, including RADtags), and has lower ascertainment bias (Sansaloni et al., 2011; Ren et al., 2015).

### Sampling a Different Number of Loci

In general, it has been suggested that if fewer populations and individuals are sampled, increasing the number of loci can increase the accuracy of estimates (Oyler-McCance et al., 2013; Peterman et al., 2016; Flesch et al., 2018; see summary and references in Table 1). In this study, we observed that after increasing the number of SNPs from 1,000, or 10 microsatellites to 5,000 (DTS), 15,000 (50K) SNPs, or 20 microsatellite loci we found similar patterns for all summary statistics and reduced variance and relative errors estimates across replicates (Figures 1–3 and Supplementary Figure S6).

These are interesting observations, since depending on the study, it may be convenient to reduce genome or microsatellite coverage to increase the number of sampled populations, especially when patterns of isolation and demographic history are analyzed. However, it is important to notice that if the objective is to find targets of selection then, increasing the number of SNPs is critical in detecting stronger neutral expectations and to reduce false positives (De Mita et al., 2013), as well as increase the probability of finding SNPs that fall within coding or regulating regions (Metzker, 2010; Ekblom and Galindo, 2011; Glenn, 2011).

### Sampling a Different Number of Individuals

We were able to compare the effect of sampling a different number of individuals for the simulated, 50K, and microsatellite datasets. For these datasets, we corroborated that sampling fewer individuals increased the variation across sampling (Miyamoto et al., 2008; Sinclair and Hobbs, 2009; Hale et al., 2012; Sánchez-Montes et al., 2017; see summary and references in Table 1), but more importantly, it underestimated the *F*_*IS*_ inbreeding estimation and overestimated the *F*_*ST*_ (Figure 1B and Supplementary Figures S2, S3, S6).

These results suggest that for genomic datasets, as long as many populations are sampled, and *H*s, *F*_*ST*_, patterns of isolation, or patterns associated to ecological variables are tested, the number of individuals is not as sensitive as the number of populations sampled covering a large portion of the distribution (Willing et al., 2012; Landguth and Schwartz, 2014). In fact, we found that it is more convenient to sample fewer individuals in as many populations as possible than the opposite (Figure 4 and Supplementary Figure S8).

On the contrary, studies that depend on *F*_*IS*_ values (i.e., genetic analyses in conservation studies; or studies that aim at detecting non-random mating), or that are performed using microsatellite data, should sample as many individuals as possible to reduce the bias generated by missing data (Flesch et al., 2018) or by identifying low frequency alleles (Supplementary Figure S7). If testing local adaptation is not a priority, then sampling fewer populations, but with many individuals (>20) might be more important, and with special focus on sampling many individuals belonging to populations that are of particular interest for the research group (i.e., threatened or vulnerable populations).

For endangered species for which fewer populations and individuals exist, if it is a priority to obtain their genetic parameters, new bioinformatics tools have been developed to estimate the demographic history based on fewer individuals sampled (Gronau et al., 2011; Schiffels and Wang, 2020). The problem is that these methods rely on large amounts of SNPs, which can be challenging to obtain if no reference genomes are available (Glenn, 2011). In such cases, it might be more important to conserve the few individuals that exist irrespective of their genetic diversity.

### Sampling a Different Number of Populations

We tested the effect of randomly sampling a different number of populations for all datasets. Sampling above 10 populations did not generate strong deviations between sampling designs and the “real” sample for the three datasets. However, we found that the number of populations was strongly associated with the accuracy and a reduction in the relative error of the mean estimates across replicates (Figures 1–3, Supplementary Figures S2–S6 and Supplementary Table S1). Sampling a different number of populations with the microsatellite dataset generated a lower variance and relative error across replicates than the genomic datasets when estimating patterns of isolation using the MRM test (Figures 2A,B and Supplementary Figure S6).

Importantly, we found that sampling fewer populations in some cases can result in opposite associations (negative instead of positive) compared to the real dataset for patterns of isolation and ecological associations (i.e., less than 10 populations for patterns of isolation, and less than 30 populations for ecological associations). Although these incorrect associations were recorded only for a few replicates (Supplementary Table S2), it is important to notice that an overestimation of false associations could result by not sampling the entire geographic and environmental distribution (see also Chao et al., 2014; Rico, 2017).

The fact that fewer populations increase variance across replicates of genomic datasets is important, because many genomic studies usually sample fewer populations in order to increase the genomic coverage (Meirmans, 2015). Our results are concordant with different studies performing simulations that have shown that increasing the number of populations increases the accuracy in estimates of summary statistics and especially in landscape genetics studies (Schwartz and McKelvey, 2009; Landguth and Schwartz, 2014; see summary and references in Table 1). In fact, we found that it is more convenient to sample more populations with fewer individuals than fewer populations with many individuals (Figure 4 and Supplementary Figure S8). Thus, we propose that if detecting local adaptation is not an objective and *F*_*IS*_ is not being measured, it is more important to sample many populations (∼30) even if fewer individuals per population are considered (Figure 4) and fewer SNPs are obtained.

### Sampling a Different Number of Populations for Detecting Outlier SNPs

An important advantage of MPS is that it allows detecting candidate loci under selection. However, an important limitation of incorrect sampling while detecting candidate loci is that demographic history and complex genetic structure can increase false positives (Schoville et al., 2012; De Mita et al., 2013; Tiffin and Ross-Ibarra, 2014). Since adaptive loci could be important for conservation (Allendorf et al., 2010) and to respond to environmental change (Bay et al., 2018; Exposito-Alonso et al., 2018; Aguirre-Liguori et al., 2019b), many efforts have been made to reduce false positives and to better detect genes that could be under selection.

When we sampled fewer populations, it was interesting to notice that mean and maximum *F*_*ST*_ values across loci were higher (Figure 5A), supporting that sampling fewer populations reduced the efficacity of estimating adequate estimates of real *F*_*ST*_ patterns, increasing the potential of false positives (De Mita et al., 2013). Interestingly, we found that as more populations were sampled and *F*_*ST*_ was estimated more accurately, more outlier SNPs were identified (Figure 5B). However, it is important to notice that the majority of the replicates did not identify the same outlier SNPs than the “real” dataset (Figure 5C and Supplementary Table S3). While this can be associated with false negatives, we rather consider that the lack of shared SNPs could correspond to the identification of outlier loci associated with different ecological settings. However, it was relevant to note that even for 30 populations, where we expect more populations to be shared with the real dataset, we still found a replicate that had only 5 shared SNPs with the entire dataset. From these analyses, we conclude that increasing the number of populations (>30) and SNPs is very important for detecting candidate SNPs since it allows the genetic structure to be defined more accurately and increases the power of the analysis (De Mita et al., 2013); and that it is important to cover the largest geographic and environmental distribution. Also, it is relevant to consider that environmental settings can have important implications on the SNPs that are identified as outliers. Our tests did not identify a strong candidate across replicates. Therefore, if genetic rescue is an objective (i.e., for conservation), it is important to perform experimental studies to corroborate the relevance of candidate SNPs (Kardos and Shafer, 2018; Bell et al., 2019).

These are important observations, especially when not many populations can be sampled either because organisms have limited distributions (Chao et al., 2014; Smith and Wang, 2014) or because there is a tradeoff between the amount of SNPs that can be obtained using MPS and the number of populations that can be genotyped (Meirmans, 2015). Methods such as *Bayescenv* (De Villemereuil and Gaggiotti, 2015), *Bayescan* (Foll and Gaggiotti, 2008), and *Bayenv* (Coop et al., 2010) do not rely on genotype counts, but rather on allelic counts. Therefore, they are not sensitive to the correct estimates of *F*_*IS*_ and one alternative can be to use a pooled sample approach to increase the number of loci and the number of populations genotyped.

## Data Availability Statement

All datasets presented in this study are included in the article/Supplementary Material.

## Author Contributions

JA-L and LEE conceived and designed the work. JA-L and JL-S performed and analyzed the genomic analyses, and wrote the first version of the manuscript. JG-P generated, performed, and analyzed the microsatellite datasets. All authors analyzed and interpreted the combined data and reviewed the manuscript.

## Conflict of Interest

The authors declare that the research was conducted in the absence of any commercial or financial relationships that could be construed as a potential conflict of interest.
